# Biomechanical Evaluation of Bone Atrophy and Implant Length in Four Implants Supporting Mandibular Full-Arch-Fixed Dentures

**DOI:** 10.3390/ma15093295

**Published:** 2022-05-04

**Authors:** Heng-Li Huang, Hui-Ling Tsai, Yu-Ling Wu, Jui-Ting Hsu, Aaron Yu-Jen Wu

**Affiliations:** 1 School of Dentistry, China Medical University, 91 Hsueh-Shih Road, Taichung 40402, Taiwan; henleyh@gmail.com (H.-L.H.); jthsu@mail.cmu.edu.tw (J.-T.H.); 2 Department of Bioinformatics and Medical Engineering, Asia University, Taichung 41354, Taiwan; 3 Department of Dentistry, Buddhist Tzu Chi Medical Foundation, Dalin Tzu Chi Hospital, No. 2, Min-Sheng Road, Dalin Town, Chiayi 62247, Taiwan; flowerelpis@gmail.com; 4 Department of Dentistry, Kaohsiung Chang Gung Memorial Hospital, College of Medicine, Chang Gung University, Kaohsiung 833, Taiwan; oxalis76@cgmh.org.tw

**Keywords:** bone atrophy, implant length, dental implants, all-on-four treatment, in vitro test, finite element method

## Abstract

Residual alveolar ridge resorption often occurs after tooth extraction, which causes issues requiring further prothesis rehabilitation. A treatment concept referred to as all-on-four, involving fixed dentures supported with four implants, was recently developed. The current study aimed to determine the effect of changing bone atrophy and implant length in all-on-four treatments on stress and strain in the surrounding bone of the implant. A three-dimensional finite element method was used in this research. The stress analysis was conducted with von Mises stress values. Two types of synthetic jawbone models with mild and moderate atrophy were used. Furthermore, two different implant lengths with a similar implant design and diameter were selected, and they were classified into eight models. Then, the bone model was assessed via a computed tomography (CT) scan and was transformed into a virtual model in Geomagic and SolidWorks with implant rebuilding. After modifying bone atrophy, the von Mises stresses in the surrounding bone of the implant were as follows: mild type 2 < mild type 3 < moderate type 3 < moderate type 4. The bone quantity change rate increased more than when bone conditions were limited. Compared with changes in implant lengths, the stresses in the peri-implant surrounding bone were generally higher in the 9 mm implant length group than in the 11.5 mm group. However, the results did not significantly differ. In conclusion, the von Mises stress and strain increased in the models with moderate atrophy and low-density trabecular bone. Hence, bone atrophy and its presurgical diagnosis in long-term implant prognosis are crucial.

## 1. Introduction

Residual alveolar ridge resorption commonly occurs after tooth extraction [1]. Severe atrophy of the alveolar ridge often results in issues requiring further prosthesis rehabilitation. Treatment success may be influenced by the size of the remaining edentulous ridges, not only the area of the denture-bearing surface but also the bone quantity and quality for the placement of the dental implant [2].

Due to advancements in dental implant surgery, there are several treatment strategies for prosthesis rehabilitation in fully edentulous ridges. These include implants retained over-dentures and implants supported by full-arch fixed dentures. These strategies follow either augmentation of the bone or the utilization of the remaining bone. In 2003, a treatment concept referred to as all-on-four was developed, which utilizes fixed dentures with four supporting implants [3]. According to a cohort study, compared with potential modalities of conventional implant treatment, the all-on-four treatment reduces treatment complexity and increases patient comfort in some cases of severe atrophy of the alveolar ridges [4]. This concept can prevent harm to significant anatomical landmarks, like the inferior alveolar nerves and maxillary sinuses. Moreover, tilted implants reduced the lengths of denture cantilevers and increased the length of implants embedded in the bone, which enlarged the bone-to-implant contact area. A previous study reported good long-term success rates and clinical performances [5]. However, another study evaluated peri-implant marginal bone loss continually for several years [6]. The occurrence of marginal bone loss was often affected by poor oral hygiene and some biomechanical factors [7,8]. The latter can relate to several factors, such as implant design, length, and diameter; bone condition; occlusal force; and medical conditions.

The bone condition is a factor influencing the long-term success of implant treatment. Based on previous studies, several scales have been used to define bone conditions. Catwood and Howell presented a scale with six different classifications of alveolar atrophy based on bone quantity [9]. By contrast, Leckhom and Zarb reported a scale with four types of bone atrophy according to bone quality [10]. In particular, the quantity of ridge resorption influenced the size of implants, and the quality of bone affected its load-bearing capacity. Multiple studies have revealed that poor bone quality may be related to lower implant success rates. Jaffinet al. reported that 3% of Branemark System implants placed in type I, II, and III bones were lost after 5 years. Meanwhile, in type IV bone, the implant failure rate after 5 years was 35% [11]. Similarly, Van Steenberghe et al. revealed that the implant failure rates were higher in the maxilla, which had poor bone quality. Since the implant surrounding the bone reacts to stresses and strains under occlusal loading, bones with poor quality may fail to overcome these loads [8].

Implant length is another factor that may influence the long-term success rate. The primary stabilization of a dental implant is an important factor influencing further implant success. Hence, inadequate primary implant stability might result in failed osseointegration [12,13]. Increasing the implant length can enlarge the surface area of an implant. However, the previous studies about the association between primary implant stability and implant length have different results. Hong et al. found that the primary implant stability was influenced by implant length [14]. By contrast, Degidi et al. reported a weak relevance between primary implant stability and implant length [15].

Since finite element method (FEM) is frequently used to explore the biomechanical effects in medical studies [16,17,18], several authors investigated the biomechanical performance of dental implants with the all-on-four treatment under three-dimensional (3D) FEM [19,20].Ozge Dognap and ErdemKilic compared the stresses transmitted to short, tilted, vertical implants and the surrounding bone in the atrophic mandible with 3D FEM [21].Kelkar et al. reported an FEM analysis on the influence of framework materials in the all-on-four implant treatment [22].However, few studies have investigated the impact of different bone atrophy conditions, including the degree of bone quantity and quality or the implant lengths on the biomechanical performance of all-on-four treatment. Therefore, the aim of the current study is to determine the effect of different bone atrophy conditions and different implant lengths in the all-on-four treatment on stress and strain in the surrounding bone of the implant under 3D FEM analysis.

## 2. Materials and Methods

Two types of synthetic jawbone models (#8571 and #8570 Synbone, Malans, Switzerland) with mild and moderate atrophy were used in this study. The two bone models were evaluated via dental computed tomography (CT) scan (PlanmecaPromax 3D Max, Planmeca, Helsinki, Finland), and a series of CT scan images were acquired and imported into medical imaging software (Mimics version 15.0, Materialise, Leuven, Belgium) to create the two 3D bone models.

The bone model comprised a dense outer layer that replicated the cortical shell and a softer inner content that limited the cancellous bone. Two types of implant lengths (9 and 11.5 mm) with a similar implant system (NobelSpeedy™ Groovy, Nobel Biocare, Goteborg, Sweden) and implant diameter (4 mm) were selected for the analysis. Moreover, abutments including straight abutments (Multi-unit Abutment, Nobel Biocare) and 30° abutments (30 Multi-unit Abutment, Nobel Biocare) used with this implant system were prepared. After a detailed measurement of implant, abutment, and customized framework dimensions, all 3D models were constructed using computer-aided design software (SolidWorks 2017, SolidWorks Corporation, Concord, MA, USA). Then, the two types of bone models were imported into the computer-aided design software with Boolean operations, and all 3D models were assembled for analysis.

The implant positions in the mild and moderate atrophy bone models were similar. Two implants were embedded into the incisor area and the other two implants were placed in the molar region. The cantilever length of the customized framework was about 3 mm long.

### Three-Dimensional FEMmodeling

After 3D modeling was completed, all the experimental models were exported to the commercial FEM software. In this step, the bone models were sliced into four major segments, thereby generating different mesh densities. The location closest to the determined location of the density of the mesh was more compact. The fixed support included both sides of the temporomandibular joint and covered parts of the mandibular ramus, which considered the anatomic construction and muscle trend (lateral pterygoid, medial pterygoid, masseter, and temporal muscle). Next, the material properties were assigned to corresponding parts, representing the incisor, canine, and second molar masticatory forces applied in the framework. Eventually, the von Mises stresses in the cortical and trabecular bones were obtained. The process was conducted in FEM software (ANSYS, ANSYS Workbench 17.2, ANSYS Inc., Huston, PA, USA).

All models in this study were meshed by a 3D tetrahedral element. The element size affects FEM results with smaller elements creating higher accuracy but taking up more computer resources and difficulties in solving. To solve this problem, the mesh quality of the FEM model was carefully arranged, and the sizes of elements from the bone area, including four implants to the temporomandibular joint were from 0.6 mm to 2.0 mm. Therefore, the position of interest in this study could get more accurately analyzed results.

**Figure 1:**
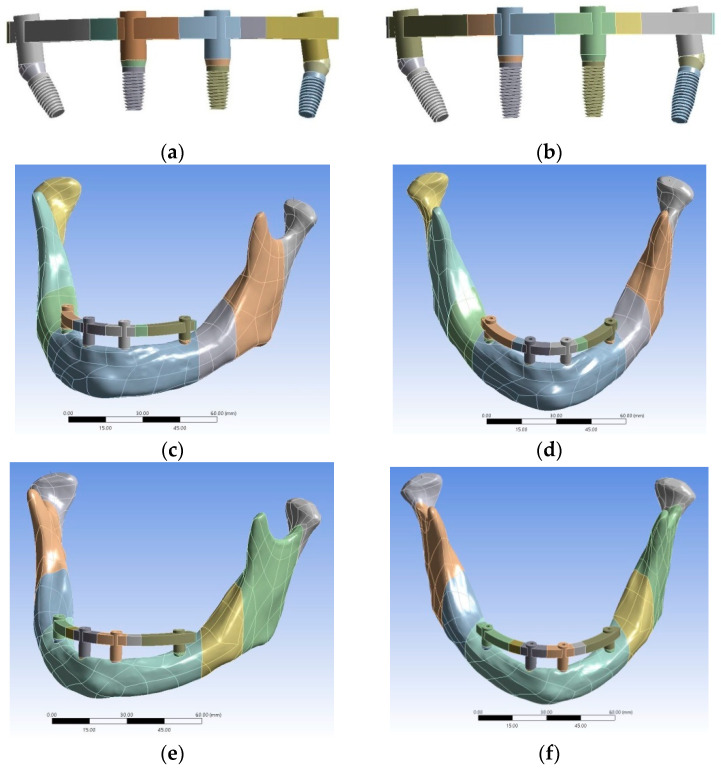
(**a**) 9 mm implant length; (**b**) 11.5 mm implant length; (**c**,**d**) mild atrophy trabecular bone; (**e**,**f**) moderate atrophy trabecular bone.

Table 1 shows the material properties of all components [23,24,25]. In this study, there were four bone conditions for simulation. Models 1 and 2 had mild atrophy (#8571), and the Young’s modulus of the trabecular bones were types 2 and 3. Models 3 and 4 had moderate atrophy (#8570), and the Young’s modulus of the trabecular bones were types 3 and 4. Thus, the bone can deteriorate sequentially from the first stage with the best bone quality (Model 1, #8571 with type 2 bone) to that with the worst bone quality (Model 4, #8570 with type 4 bone). Moreover, #8571 with type 3 bone to #8570 with type 3 bone can be considered as the transitional stage, which is linked to the association between mild and moderate bone atrophy. Meanwhile, Models 1 and 5 had similar bone conditions (mild atrophy type 2 trabecular bone) but different implant lengths (Model 1: 9 mm; Model 5: 11.5 mm) (Figure 1). Models 2 and 6 presented with mild atrophy type 3 trabecular bone but different implant lengths (Model 2: 9 mm, Model 6: 11.5 mm). Models 3 and 7 had moderate atrophy type 3 trabecular bone but different implant lengths (Model 3: 9 mm, Model 7: 11.5 mm). Models 4 and 8 presented with moderate atrophy type 4 trabecular bone but different implant lengths (Model 4: 9 mm, Model 8: 11.5 mm).

**Figure 2:**
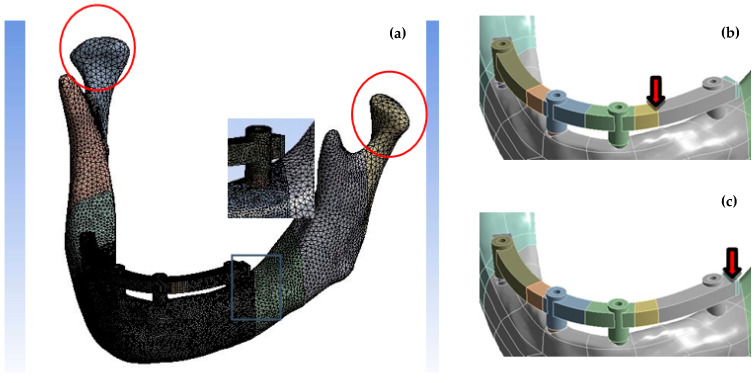
(**a**) The finite element model; the red circles represent the regions of boundary condition. (**b**) Loading condition A and (**c**) Loading condition B are shown.

The materials of implants, abutments, frameworks, and bone were homogeneous and had isotropic elastic properties. The interface between the bone and implant was set as boned-based on the assumption of 100% osseointegration. The boundary conditions of the FEM models were set to fix the surface of the condyle to zero displacement in three directions. Two types of loading conditions were tested (Figure 2). Loading condition A was a point vertical load of 65N applied to the premolar region of the framework. Loading condition B was also a vertical force of 150 N applied to the second molar area of the framework, which is also near the end area of the framework. After the FEM solution, the observation focused on the von Mises stress of the cortical bone near the implants of each model.

## 3. Results

### 3.1. Bone Atrophy versus Von Mises Stresses

**Figure 3:**
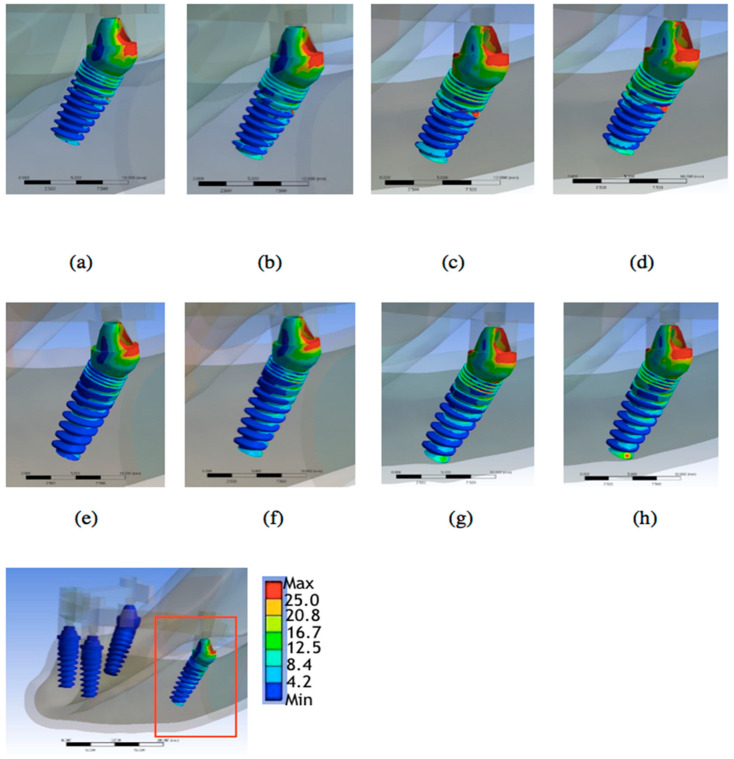
Dental implant with a high stress distribution in (**a**) Model 1, (**b**) Model 2, (**c**) Model 3, (**d**) Model 4, (**e**) Model 5, (**f**) Model 6, (**g**) Model 7, (**h**) Model 8.

**Figure 4:**
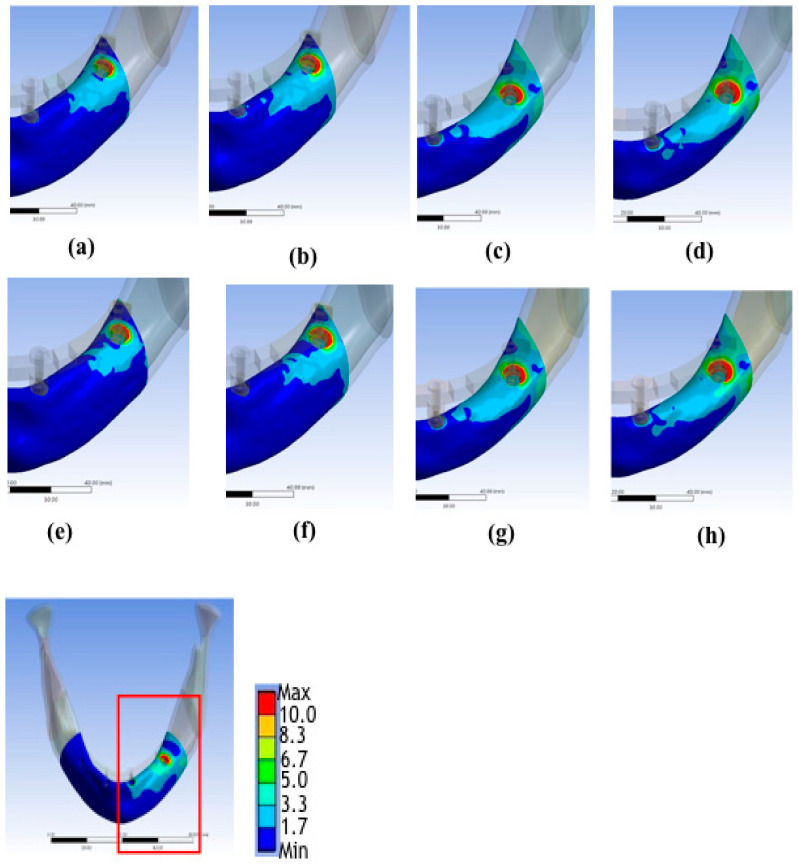
Distributions of von Mises stresses in the cortical bone in (**a**) Model 1, (**b**) Model 2, (**c**) Model 3, (**d**) Model 4, (**e**) Model 5, (**f**) Model 6, (**g**) Model 7, (**h**) Model 8.

**Figure 5:**
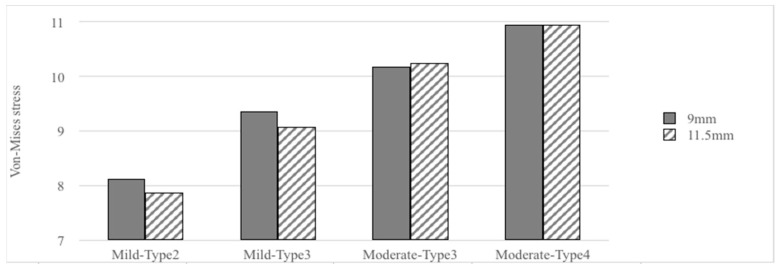
Peak values of von-Mises stresses in the cortical bone in loading condition A.

**Figure 6:**
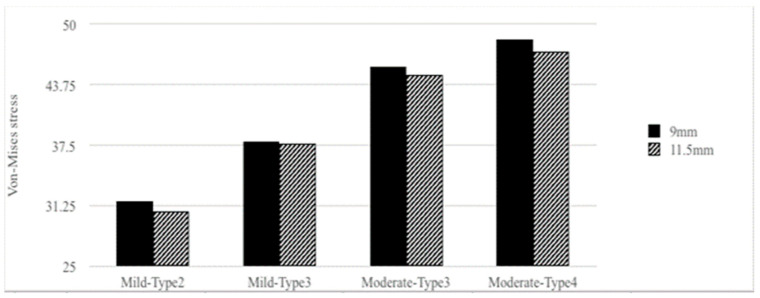
Peak values of von-Mises stresses in the cortical bone in loading condition B.

Table 2 and Figure 3, Figure 4, Figure 5 and Figure 6 show the results. There were four bone atrophy conditions (mild type 2, mild type 3, moderate type 3, and moderate type 4). Overall, with similar implant lengths and loading conditions, the von Mises stresses in the surrounding cortical bone around the implant were as follows: mild type 2 < mild type 3 < moderate type 3 < moderate type 4. The stress in the surrounding cortical bone around the implant was the highest in moderate type 4 with an implant length of 9 mm under loading condition B (48.44 MPa), and it was slightly higher than similar bone and loading conditions with an implant length of 11.5 mm (47.21 MPa).

When focusing on stresses in the surrounding cortical bone with bone quality change, mild type 3 was 19.42% higher than mild type 2 in the 9 mm implant length group under loading condition B. Furthermore, moderate type 4 was 6.08% higher than moderate type 3. Similarly, mild type 3 was 22.59% higher than moderate type 3 in the 11.5 mm implant length group under loading condition B. Further, moderate type 4 was 5.35% higher than moderate type 3. Therefore, if the bone quality decreased, the von Mises stresses increased (type 2 < type 3 < type 4).

The effect of changing bone quantity on the stress in the surrounding cortical bone around the implant was also considered. In the current study, moderate type 3 was 20.37% higher than mild type 3 in the 9 mm implant length group under loading conditions. Similarly, moderate type 3 was 18.98% higher than mild type 3 in the 11.5 mm implant length group under loading condition B. The results showed that the vonMises stresses increased (mild < moderate) whenthe bone quantity decreased.

However, the change in bone quality or quantity, which played a more important role, remained uncertain. Compared with the von Mises stresses in the surrounding cortical bone around the implant, moderate type 3 was 20.37% higher than mild type 3 in the 9 mm implant length group under loading condition B. Further, moderate type 4 was 6.08% higher than moderate type 3. The bone quantity change had a higher increased rate than the bone quality change if bone atrophy was limited (Table 2, Figure 4, Figure 5 and Figure 6).

### 3.2. Implant Lengths versus Von Mises Stresses

Under similar bone conditions, the stresses in the cortical bone were generally higher in the 9 mm implant length group than in the 11.5 mm implant length group. However, the result did not significantly differ. That is, in mild type 2 bone conditions, the von Mises stress in the cortical bone around the implant in the 11.5 mm implant length group was 3.17% lower than that in the 9 mm implant length group under loading condition A. Similarly, it was 3.38% lower in the 11.5 mm implant length group than in the 9 mm implant length group under loading condition B.

By contrast, with different bone conditions and similar loading conditions, the rate was higher in different bone conditions than in various implant lengths. Considering the von Mises stresses in the cortical bone around the implant under loading condition B, the value was 3.38% lower in the 11.5 mm implant length group than in the 9 mm implant length group with mild type 2 bone condition. Moreover, the value was 2.60% lower in the moderate type 4 bone condition. However, in the 9 and 11.5 mm implant length groups, the mean stress was 53.07% higher in the moderate type 4 bone condition than in the mild type 2 bone condition under loading condition B.

Consequently, the effect of bone condition might be higher than the effect of implant lengths. However, the result might be attributed to the fact that the difference in implant length (9 vs. 11.5 mm) was minimal. Nevertheless, further studies should be conducted to obtain better conclusions (Table 2, Figure 4, Figure 5 and Figure 6).

### 3.3. Loading Conditions versus Von Mises Stresses

Generally, the von Mises stresses in the cortical bone around the implant under loading condition B were significantly higher than those in loading condition A. When calculating the mean volume of the stresses of 9 and 11.5 mm implant lengths to disregard differences in implant length, the stress under loading condition B was 290.98% higher than that under loading condition A in the mild type 2 bone condition. Thus, regardless of changes in bone conditions and implant lengths, the stress in the cortical bone around the implant was the highest under loading condition B.

Moderate atrophy of type 4 bone had the worst biomechanical performance under loading condition B (Table 2, Figure 4, Figure 5 and Figure 6).

## 4. Discussion

Alveolar ridge resorption after edentulous ridge, particularly severe atrophy alveolar ridge, is a challenge in full mouth reconstruction. Due to the development of dental implant surgery, there were more treatment options for the full edentulous ridge. The concept of placing five to six endosseous implants in the intermental region with a fixed bridge was developed by the Branemark group [6,26]. It was considered a reliable treatment option and implant survival rates were high at 90%–98%. To date, the concept of the all-on-four treatment for full mouth reconstruction of fully edentulous ridges has gained in popularity. Several studies have performed FEM analysis, thereby making it easier to investigate the peak values and the distribution of bone stresses and strains [27]. Animal experiments [7,28] and clinical studies [6,8] reported that peri-implant marginal bone loss might lead to implant failure related to unfavorable loading conditions. Improper loading might cause excessive stress in the surrounding bone of implants, which results in bone resorption. Hence, the stresses and strains in the implant surrounding bone and their relevance to different parameters of the implants and bones must be investigated. The current study used the FEM analysis to determine the effect of different bone conditions and implant lengths with the all-on-four treatment on stress in the implant fixture and the peri-implant surrounding bone.

### 4.1. Effect of Bone Atrophy

The quality and quantity of bone atrophy were considered. According to the literature review studies, bone quality influences implant success. Clinical studies showed that implants placed in type I and type II bones (bone quality classification by Lekholm and Zarb) [10] had a good long-term prognosis. However, in implants placed in unfavorable bone types, particularly type IV bone, the failure rates were high [11]. Bone density may affect implant failure, and the elastic modulus is based on the density or porosity of the bone [29]. Thus, the Young’s modulus of the bone was changed to evaluate its influence on stress and strain in the implant and surrounding bone. Furthermore, bone quality is affected by other factors such as trabecular bone architecture and amount of cortical bone, which were not discussed in this study.

A low-density trabecular bone shows low stiffness, which may provide less support for dental implants. This phenomenon may also lead to a greater burden in the cortical bone such as increased stress and strain. This result could explain the findings of clinical reports showing that type IV bone had higher implant failure rates than type I–III bones [8,11]. However, few studies have considered bone quantity. In the study of Lopes et al., patients were classified as follows: surgical difficulty in scoring as low (residual ridge > 5-mm wide), moderate (residual ridge that is 4–5-mm wide), or high (residual ridge < 4-mm wide) [30]. Furthermore, Tallarico et al. revealed that the Cawood and Howell classification could be an indication criterion, considering discrepancies in resorption degree. The current study found that the all-on-four treatment is a reliable and effective technique for jaws in patients with class IV, V, and VI bones, based on the Cawood and Howell classification system [31]. In this research, the peak values of von Mises stress increased with greater severity in bone atrophy and lower bone density (Figure 5 and Figure 6). The results were similar to those of previous review studies. Furthermore, bone quantity change had higher von Mises stress than if bone atrophy was limited. Nevertheless, further studies must be conducted to obtain specific conclusions.

### 4.2. Effect of Implant Lengths

Implant length was another factor influencing implant success. Van Steenberghe et al. reported that implant failure rates were 10.7% in 7 mm implants and approximately 5.9% in 10 and 13mm implants in the maxilla. Meanwhile, none of the implants with a length of 15 mm or longer failed [8]. The length had no impact on the success rate in good-quality bones. Likewise, in our study, the implant had a greater effect on the peak von Mises values in moderate atrophy trabecular bone types 3 and 4 under loading conditions, compared with mild atrophy trabecular bone types 2 and 3 (Figure 5 and Figure 6).

In the current study, there were no significant differences between the 9 mm implant length group, which had higher peak values of von Mises stresses under loading conditions, and the 11mm implant length group. However, this result may be associated with minimal changes in implant length (9 versus 11.5 mm). Thus, these results might be associated with reports showing that bone conditions and implant lengths affect implant success. The lower strain found in longer implants might result from a larger bone-implant contact area, which added resistance during implant displacement.

### 4.3. Effect of Loading Conditions

Several studies showed that cantilevers increased the risk of overloading in implant-supported prostheses [32,33]. Rodriguez et al. revealed that longer cantilevers contributed to higher stress at implant sites, thereby inducing greater marginal bone loss around implants [34].

Similarly, in our study, loading condition B might be the worst loading condition as it had the highest von Mises stress in the surrounding bone of the implant. As presented previously, loading condition B was applied at a vertical load of 150 N to the framework in the second molar area. Therefore, it was used as a vertical load to the cantilever area of the framework.

### 4.4. Limitations

Some assumptions were adopted in our study to simulate bone conditions. Specifically, the ideal and unrealistic conditions of complete osseous integration were surmised. Higher stress values were caused by a lower degree of osseous integration. Moreover, static vertical forces alone, without inclusion of horizontal and oblique vectors of occlusal forces, were applied. The simply assumed boundary condition of fixed support differed from realistic muscle–jaw interactions [35]. Bones, implants, and frameworks were modeled as dry isotropic linear elastic materials, assuming that mechanical properties are time-independent. Moreover, the occlusal surface was simply assumed as the top surface of the framework without considering the tooth morphology.

These assumptions did not completely represent clinical practices due to possible osseous integration defects at the peri-implant regions; different loading distributions between patients; more complicated and time-dependent forces and the impact of muscles; and anisotropic [36], non homogeneous, nonlinear, and inelastic response of living tissue properties. Nevertheless, in agreement with other numerical studies [37,38], the currentassumptions are acceptable, in a computational sense, to deduce significant and clinically useful indications in all-on-four treatment studies.

## 5. Conclusions

Within the limitations of this research, the conclusions show that, first, the von Mises stresses in the cortical bone around the implant were higher under loading conditions that applied a vertical load to the cantilever area of the framework than under loading conditions that applied a vertical load to the non-cantilever area. Second, for investigating the effect of mandibular bone atrophy, if the bone quality decreased, the von Mises stresses increased (type 2 < type 3 < type 4). By contrast, if the bone quantity decreased, the von Mises stresses increased (mild < moderate). Furthermore, the rate was higher in bone quantity change than in bone quality change if bone atrophy was limited. Third, for different implant lengths, the stresses in the cortical bone were generally higher in the 9 mm implant length group than in the 11.5 mm implant length group. However, the results did not significantly differ. Moreover, the rate was higher in different bone conditions than in various implant lengths. All in all, the von Mises stress and strain increased in the models with moderate atrophy and low-density trabecular bone, thereby confirming the importance of bone atrophy and its presurgical diagnosis in long-term implant prognosis. Last but not least, due to the limitation of in vitro study, further studies are needed to confirm the results above.

## Figures and Tables

**Table 1:** Material properties in the FEM model.

Material		Young’s Modulus E (MPa)	Poisson’s Ratio
Cortical bone		14,800	0.3
Trabecular bone	Type2	5500	0.3
	Type3	1600	0.3
	Type4	690	0.3
Titanium implant		110,000	0.35
Titanium alloy framework		110,000	0.35

**Table 2:** The peak values of von Mises stresses located on fixture of the implant as well as surrounding cortical bone around the implant at different loading conditions.

Atrophy	Trabecular Bone	Implant Length	Location of the Peak Stress	Loading Condition A (MPa)	Loading Condition B (MPa)
Mild	Type 2	9.0	Fixture	36.04	108.67
(Model 1)			Cortical bone	8.12	31.76
Mild	Type 3	9.0	Fixture	37.98	201.36
(Model 2)			Cortical bone	9.37	37.93
Moderate	Type 3	9.0	Fixture	42.50	232.61
(Model 3)			Cortical bone	10.18	45.66
Moderate	Type 4	9.0	Fixture	54.24	268.15
(Model 4)			Cortical bone	10.95	48.44
Mild	Type 2	11.5	Fixture	21.03	52.83
(Model 5)			Cortical bone	7.87	30.72
Mild	Type 3	11.5	Fixture	24.04	51.47
(Model 6)			Cortical bone	9.08	37.66
Moderate	Type 3	11.5	Fixture	40.87	62.00
(Model 7)			Cortical bone	10.24	44.81
Moderate	Type 4	11.5	Fixture	43.65	64.76
(Model 8)			Cortical bone	10.94	47.21

## Data Availability

Not applicable.
